# Determinants of anti-retroviral regimen changes among HIV/AIDS patients of east and west Wollega zone health institutions, Oromia region, west Ethiopia: a cross-sectional study

**DOI:** 10.1186/s40360-018-0220-7

**Published:** 2018-06-05

**Authors:** Amsalu Bokore, Belay Korme, Getu Bayisa

**Affiliations:** 1 Nekemte referral hospital, Nekemte, Ethiopia; 2 Wollega University, Nekemte, Ethiopia

**Keywords:** HIV/AIDS, HAART, ARV drug, Regimen change, Wollega

## Abstract

**Background:**

Human Immunodeficiency Virus (HIV) is one of the main causes of morbidity and mortality; because of this it continues to be a major global public health concern. It has believed to kill more than 34 million lives so far. Sub Saharan Africa constitutes about 70% of people living with HIV among the 37 million on the globe. This region, accounted for more than two third of the global new HIV infections and about 15 million (40%) were receiving antiretroviral therapy (ART) at the end of 2014 throught the world. ART has fundamentally changed the treatment of HIV and transformed this infection from a disease of high mortality to chronic and medically managed disease. The issues of drug induced toxicities & complexity of current highly active antiretroviral therapy (HAART) regimens has remained of great concern. The aim of this study was to determine factors leading to antiretroviral regimen changes among HIV/AIDS Patients in the study area.

**Methods:**

A facility based retrospective cross-sectional study was conducted from April 28, 2017 to May 30, 2017 in the ART clinics of east and west Wollega zone health institutions using a pre-tested data collecting form and chart review. The sample included the 243 patients whose medication had been switched.

**Results:**

Majority 145 (59.67%) of the patients had been on ART for > 10 years duration. More than half 126(51.9%) of the patients had received tuberculosis (TB) treatment and almost three out of five patients (57.2%) had received isoniazid & cotrimoxazole prophylaxis. The most common reason for regimen change was peripheral neuropathy 146(60.1%) and the most common medication for this reason was stavudine, lamivudine and neverapine based 108(44.44%).

**Conclusions:**

The number of patients who changed ARV drug in our resource constrained setting present a challenge to the restricted treatment choices that we currently own. Less toxic and better-tolerated HIV treatment options should be available and used more frequently.

## Background

Human immunodeficiency virus (HIV) annihilates and compromises the function of immune cells. Immunity of infected individuals gradually depletes and susceptibility to a wide range of infections and diseases would be boosted. Acquired immunodeficiency syndrome (AIDS) is the most advanced stage of HIV infection, which can take from 2 to 15 years to develop depending on the individual and it can be explained by the progression of opportunistic infections, or other intense clinical manifestations and certain cancers [1, 2].

HIV is one of the main causes of morbidity and mortality; because of this it continues to be a major global public health concern. It has believed to kill more than 34 million lives so far. Sub Saharan Africa constitutes about 70% of people living with HIV among the thirty seven million on the globe. This region, accounted for more than two third of the global new HIV infections in 2014 with only 12% of the global population. Globally people receiving ART were about fifteen million (40%) among those living with HIV of which about fourteen million were in low- and middle –income countries and nearly one million were children [2].

Ethiopia is among the countries most affected by HIV/AIDS with prevalence of 1.9% for women and 1.0% for men in 2011 [3] and it is also in a low generalized HIV epidemic with significant heterogeneity among regions and population groups [4]. The existence of HIV infection in Ethiopia was recognized in the early 1980s with the first two AIDS cases reported in 1986. The predominant strain is HIV-1 subtype C, predominantly spread through unprotected heterosexual intercourse [5]. Since 2000 the epidemic has declined [6] and recent figures show that HIV infection has significantly decreased over the years in the country [4].

Highly active antiretroviral therapy (HAART) has fundamentally changed the treatment of HIV and changed this infection from a disease of high mortality to chronic and medically managed disease which is a radical change in controlling the hardship of HIV/AIDS. However drug resistance and side effects were the great concern in these advancements [7]. Revolution in the care of patients with HIV/AIDS occurred due to the innovation of potent HAART in around 2000. The qualities of life of people living with HIV/AIDS (PLWHA) have improved and these treatments have dramatically reduced rates of mortality and morbidity among these patients. This result was also confirmed by World health organization (WHO) progress report. ARV drugs produce these effects by restoration of number and quality of cluster of difference (CD4) cells and suppressing viral replication [8].

The goal of ART is to attain maximal and durable suppression of the viral replication. Viral suppression enables recovery of the immune response and thereby reduces risk of opportunistic infections (OIs) and death [1].

The decision to initiate ART for adults and adolescents depend on: WHO stage 3 and 4 disease irrespective of CD4 cell count, CD4 count ≤500cells/mm^3^ irrespective of WHO clinical stage and active tuberculosis (TB) co-infection with HIV irrespective of CD4 cell count according to standard treatment guideline of Ethiopia [6].

Triple combination therapy has been in use for more than two decades globally. Currently, the preferred first regimen triple therapy in Ethiopia consists of, two Nucleoside Reverse Transcriptase Inhibitors (NRTIs) and one Protease Inhibitor (PI) or a Non-Nucleoside Reverse Transcriptase Inhibitor (NNRTI) or a triple therapy of three NRTIs. Based on the guideline, common ART regimens in the country are; Tenofovir (TDF)/ Emtricitabine (FTC)/ Efavirenz (EFV) or Nevirapine (NVP); alternatives are TDF/ Lamivudine (3TC) /EFV or NVP, Zidovudine (ZDV)/3TC/EFV or NVP. Other options are Abacavir (ABC)/3TC/NVP, ABC/3TC/EFV and ABC/3TC/ZDV. The second line regimen consists of ZDV ±3TC + Lopinavir/ritonavir (LPV/r) (or Atazanavir/ritonavir (ATV/r)), ZDV + ABC + LPV/r (or ATV/r), TDF/3TC ± ZDV + LPV/r (or ATV/r), ABC/ Didanosine (ddI) /LPV/r (or ATV/r), EFV or NVP / LPV/r (or ATV/r) [1, 9].

Changes of multiple medications in HAART regimens were commonly required simultaneously. These changes may be due to co morbidity with other chronic diseases, a desire for pregnancy, poor adherence, stock out of drugs, treatment failure, long term toxicity or acute toxicity. The approaches to change ART regimens depend largely on amount of previous ART experience, available treatment options and reason for change. For example effective treatment can be accomplished by substituting another agent for the drug which has unpleasant effect in the regimen when it develops to certain drugs in the regimen [7, 10].

The issues of drug toxicities and complexity of current HAART regimens has remained of great concern despite ARTs being of much help to the health of HIV/AIDS patients. Suboptimal therapy, discontinuation, and treatment failure can be resulted from treatment toxicities and adherence problems [11]. The consequence of these may complicate the management and lead to toxicity, loss to follow-up, compromise the effectiveness of HAART regimens, drug interactions and drug resistance [12].

Knowledge of the determinants of ART change may help to minimize the risk factors. These benefits in decreasing the rate of regimen change, treatment failure, drug resistance, and improve the quality of life of the patient. Antiretroviral treatment change should be done when necessary to spare the future treatment options. The approach to patients who need to switch will differ depending on several issues, including ART experience and available options. Regimen substitution requires adjustment in learning the new medication about the treatment dosing, time of intake and deal with many individual based inconveniences, which might be challenging and reason for non-adherence [10, 13].

Data on modification of HAART and factors associated with ARV drug regimen change are limited among HIV/AIDS patients in Ethiopia. Most of the surveys used were on small sample of patients who were on ART for less than three years. As a result it is important to understand common reasons of ARV drug switch in patients on long period exposure to ART.

Therefore, this study attempts to investigate the major determinants of HAART regimen change among HIV/AIDS patients in east and west Wollega zone health institutions by using cross-sectional study.

## Objective

### General objective

- ✓ To assess determinants of antiretroviral regimen change among HIV/AIDS patients in east and west Wollega zone health institutions, Oromia region, west Ethiopia.

### Specific objectives

- ✓ To assess determinants of antiretroviral regimen change among HIV/AIDS positive patients.
- ✓ To identify the pattern of initial ART regimens and the subsequent changes.
- ✓ To assess the relationship between patient characteristics and reasons for initial ART change.

## Methods

### Study area, design, and period

The study was conducted in the ART clinics of east and west Wollega zone health institutions, Oromia region, west Ethiopia; Nekemte town which is the capital of east Wollega is located 328 km where as Gimbi town which is the capital of west Wollega is 438 Km western to Addis Ababa [14].

The area is well known by its coffee production. The economy of the people is based on subsistence farming and livestock rearing. The climatic condition of the area is ‘woinadega’ (semi-desert) and it is found at 2080 m above sea level.

A facility based retrospective cross-sectional study was conducted by reviewing patient information sheets and physician diagnostic cards to assess reasons for HAART regimen change. The study was conducted from April 28, 2017 to May 30, 2017.

### Source and study population

All HIV/AIDS positive patients who were greater than 18 years and on HAART in east and west Wollega zone health institutions ART Clinic from April 28, 2007 to April 28, 2017 were the source population. All HIV/AIDS positive patients greater than 18 years who had undergone HAART regimen change in east and west Wollega zone health institutions ART Clinic in between April 28, 2007 to April 28, 2017 were the study population.

### Inclusion and exclusion criteria

Inclusion criteria:

- ✓ Patients on follow up in the ART clinic who had undergone HAART regimen change until the study period.
- ✓ Patients on follow up in the ART clinic who were on second line regimen when the study was undergone.
- ✓ HIV/AIDS patients who were greater than18 years.
- ✓ Patients receiving HAART regimen for at least 6 months at the beginning of the study period

Exclusion criteria:

- ✓ Patient information cards with incomplete information (Patient information card which had no one or more of information like information on demographics, WHO clinical stage, CD4 count, initiation regimen and changed regimen, duration of initial therapy, and causes for regimen change).
- ✓ Patients with less than 6 months on HAART regimen.
- ✓ Patients who didn’t switch HAART regimen.
- ✓ Under eighteen year old HIV/AIDS patients.
- ✓ Deceased patients
- ✓ Transfer out patients

### Sample population

A total of 243 patients who had undergone HAART regimen change in the ART clinics of east and west Wollega zone health institutions from April 28, 2007 to April 28, 2017 were included in the study while patients below 18 years were excluded from the study. Patient information cards that showed a change in the initial treatment regimen were assessed and analyzed, to identify the common reasons that resulted in a change from the initial treatment regimen.

### Study variables

**Independent variables**

- ✓ Socio-demographic characteristics: age at initiation, sex, marital status, educational status.
- ✓ Disease related variables: baseline WHO stage, base line CD4, and baseline weight.
- ✓ ART related variables: types of initial regimen

**Dependent variables**

- ✓ Reasons for change

### Data procedure and management

#### Data collection procedure

Data abstraction form was developed based on the objectives of the study. It contained socio-demographic, clinical information and ART information such as, CD4 count, WHO stage, initial regimen, date on which treatment was started, date of ARV drug switch, duration of initial ARV therapy before first switch, regimen switched to, and causes for regimen change. The types of toxicity and treatment failure reasons were included. If there was ARV drug switch for the second and third time it was recorded in a similar manner. For data collection four 10th grade completed students were recruited. One pharmacist and one druggist from each health institutions were also recruited as supervisors.

### Data collectors recruitment and training

Data collectors were recruited and trained methods of data collection prior to the start of actual data collection.

### Data quality assurance/control

Training was provided for supervisors and data collectors and they were standardized. Data abstraction form was pre-tested on randomly selected patient information cards to identify any drawbacks in Shambu hospital which is found in Horro Gudru Wollega zone before the actual survey and improvements were made. The principal investigator supervised the data collection. Every questionnaire was checked for completeness and logical consistency.

### Data processing and analysis

The data were coded and entered in to a computer using statistical Package for the Social Sciences (SPSS) software for windows version 20 and the analysis was performed after the data were cleaned, edited and processed. Distribution of Patients such as percentages and their number by socio demographic characteristics and other relevant variables in the study were described using descriptive analysis.

### Ethical considerations

An official letter was written by department of pharmacy, college of public health and medical sciences, Wollega University to zonal and woreda health offices of east and west Wollega zone Administration to get permission. After permission to conduct the study was obtained, data has been collected in one of refilling rooms at ART Clinic by safe keeping of records.

Only numerical identifications were used as a reference, confidentiality and anonymity of subject was maintained by not recording and identifying details, such as name or any other personal details. No disclosure of any name of the patients, the healthcare provider or drug product was made in relation to the finding.

### Operational definitions

**ABC based regimen**: regimen containing abacavir as one of the NRTI backbones and may have different NNRI or PI bases.

**Antiretroviral drug switch/change**: it is the change of one or two ARV drugs from the initial drug regimens.

**AZT based regimen**: regimen containing zidovudine as one of the NRTI backbones and may have different NNRI or PI bases.

**Co morbidity**: is defined as the occurrence of one or more additional disorders which are on drug therapy with HIV/AIDS simultaneously (TB, diabetes, hypertension).

**d4T based regimen**: regimen containing Stavudine as one of the NRTI backbones and may have different NNRI or PI bases.

**TDF based regimen**: regimen containing Tenofovir as one of the NRTI backbones and may have different NNRI or PI bases.

**Toxicity**: is defined as the occurrence of adverse events such as diarrhea, nausea, vomiting, anemia, rash, fatigue, peripheral neuropathy, lipodystrophy, metabolic disturbances, CNS abnormalities or any other unwanted effect related to HAART.

**Transfer out:** Patients who changed their follow up to other health institution.

## Results

### Socio-demographic characteristics of patients whose ART regimen changed

The mean age of patients was 43.68(SD ± 8.2) years. Majority of the patients 174(71.6%) were in the age range of 35–49 years and more than half 127(52.3%) of the patients were females. About 154(63.4%) of the patients were married. In this study only 77(31.7%) of the patients received greater than secondary school education. Regarding the family size 96(39.5%) of the patients had < 5 family size whereas 61(25.1%) had > 10 family size. Regarding place of residence 209(86%) were urban dwellers where as 34(14%) were rural dwellers as shown in (Table 1).

**Table 1:** Socio-demographic characteristics of HIV/AIDS patients who changed their HAART regimen in east and west Wollega zone health institutions, April 28, 2007 to April 28, 2017

Demographic Characteristics	N (%)
Age in years	
20–34	19(7.8)
35–49	174(71.6)
≥ 50	50(20.6)
Sex	
Female	127(52.3)
Male	116(47.7)
Marital status	
Single	18(7.4)
Married	154(63.4)
Divorced	12(4.9)
Widowed	59(24.3)
Educational status	
No formal education	74(30.5)
Primary school education	92(37.9)
Secondary school education	52(21.4)
Higher institute education	25(10.3)
Family size	
Less than five	96(39.5)
5–10	86(35.4)
> 10	61(25.1)
Place of Residence	
Urban	209(86)
Rural	34(14)

### Clinical characteristics of patients whose ART regimen was changed

**Fig. 1:**
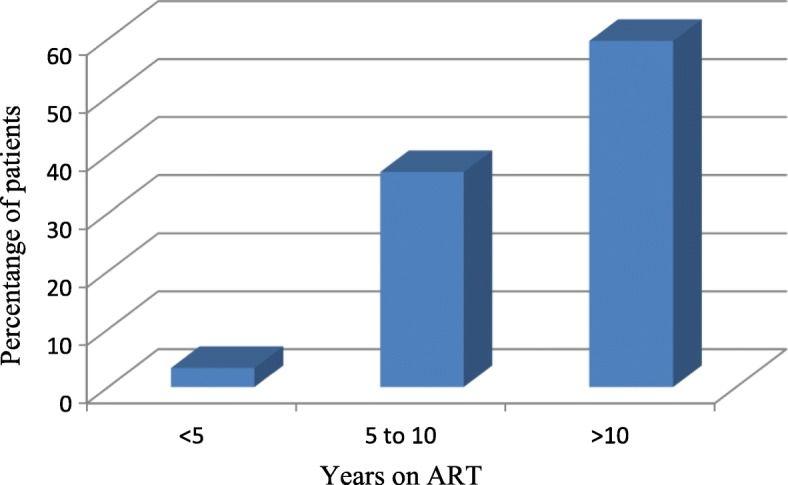
Years of stay on ART of HIV/AIDS patients who changed their HAART regimen in east and west Wollega zone health institutions, April 28, 2007 to April 28, 2017

During initiation of ART 180(74.1%) of the patients were on WHO clinical stage III; whereas WHO stage after the ART switch was stage I for 237(97.53%) of patients. Similarly more than two third 180(74.1%) of patients had baseline CD4 count less than 200 cells/μL on initiation but most 190(78.19%) of patients had > 350 cells/μL after ART switch. In addition, the weight of the majority 164(67.5%) of patients during initiation of ART was between 45 and 60 kg (Table 2). Majority 145 (59.67%) of the patients had been on ART for > 10 years duration (Fig. 1). About 126(51.85%) of the patients had received TB treatment whereas 117(48.15%) of the patients did not receive TB treatment. Regarding OI prophylaxis 139(57.2%) of the patients received Cotrimoxazole and Isonazid prophylaxis (Table 3).

**Table 2:** Clinical characteristics of HIV/AIDS patients who changed their HAART regimen in east and west Wollega zone health institutions, April 28, 2007 to April 28, 2017

		On initiation of ART	Before ART switch	On data collection
WHO clinical stage	stage I	17	60	237
	stage II	40	55	6
	stage III	180	126	0
	stage IV	6	2	0
	Total	243	243	243
CD4 count (cells/ml)	< 200	180	42	27
	200–350	60	54	26
	> 350	03	147	190
	Total	243	243	243
Weight (kg)	< 45	51	24	24
	45–60	164	155	137
	> 60	28	64	82
	Total	243	243	243

**Table 3:** OI prophylaxis taken by study population in east and west Wollega zone health institutions, April 28, 2007 to April 28, 2017

Type of OI prophylaxis	Frequency	Percent
Cotrimoxazole & Isoniazid	139	57.2
Cotrimoxazole	95	39.1
Neither	9	3.7
Total	243	100.0

### Patterns of initial ART regimen and regimen switched to/changed regimen

Majority of the patients 159(65.4%) started their initial ART on D4t-3TC-NVP regimens, followed by D4t-3TC-EFV 55 (22.6%). Only 12% of patients started initial ART regimen on AZT based regimen (Table 4).

**Table 4:** HAART regimen at initiation among HIV/AIDS patients who changed their HAART regimen in east and west Wollega zone health institutions, April 28, 2007 to April 28, 2017

Initial HAART regimen	Frequency	Percent
D4t-3TC-NVP	159	65.4
D4t-3TC-EFV	55	22.6
AZT-3TC-NVP	14	5.8
AZT-3TC-EFV	15	6.2
Total	243	100.0

The HAART regimen of majority of the patients 168(69.14%) was changed to TDF based regimen. Whereas about 69(28.4%) of the patients initial HAART regimen was changed to AZT based regimen and only 6(2.5%) patients initial HAART regimen was changed to ABC based regimen (Table 5).

**Table 5:** Patterns of ART switch of HIV/AIDS patients who changed their HAART regimen in east and west Wollega zone health institutions, April 28, 2007 to April 28, 2017

ART drugs after switching	Frequency	Percent
AZT + 3TC + NVP	47	19.3
AZT + 3TC + EFV	16	6.6
TDF + 3TC + EFV	56	23.05
TDF + 3TC + NVP	98	40.33
ABC+ ddi + LPV/R	6	2.5
AZT + 3TC + ATV/R	6	2.5
TDF + 3TC + LPV/R	10	4.1
TDF + 3TC + ATV/R	4	1.6
Total	243	100.0

### Reasons for ART change

The main reason for antiretroviral regimen change was Peripheral neuropathy 146(60.1%) followed by hepatotoxicity, d4t faith out, CNS toxicity, Anemia, Rash etc. (Table 4). The most common reason for regimen change was peripheral neuropathy 146(60.1%) and the most common medication for this reason was stavudine, lamivudine and neverapine based 108(44.44%) (Table 6 and 7).

**Table 6:** Common reasons for modification of regimens of study population in east and west Wollega zone health institutions, April 28, 2007 to April 28, 2017

Reason for regimen change	Frequency	Percent
Peripheral neuropathy	146	60.1
Hepatotoxicity	22	9.1
d4t phase out	18	7.4
CNS toxicities	16	6.6
Anemia	16	6.6
Rash	15	5.3
Others	10	4.9

**Table 7:** Common reasons for modification by first treatment regimens among study population in east and west Wollega zone health institutions, April 28, 2007 to April 28, 2017

Patterns of ART Regimen	Reasons for ART regimen change										Total
	Anemia	Rash	Peripheral neuropathy	Hepatotoxicity	Diarrhea	CNS toxicities	stigma disclosure	d4t phase out	jaundice	Burning/numbness	
d4t-3TC-NVP	5	8	108	16	0	4	0	16	0	2	159
d4t-3TC-EFV	0	3	30	6	0	10	2	2	0	2	55
AZT-3TC-NVP	4	0	2	0	4	2	0	0	2	0	14
AZT-3TC-EFV	7	2	6	0	0	0	0	0	0	0	15
Total	16	13	146	22	4	16	2	18	2	4	243

## Discussion

The present retrospective cross sectional study of HIV/AIDS patients who changed their HAART regimen in east and west Wollega zone health institutions described the pattern of ART regimen and the common reasons for ARV drug switch. Such studies would be helpful in understanding the complexity of ART use of patients in health institutions which might have different co morbidities.

Majority of the study population 145 (59.67%) had been on ART for > 10 years. This finding indicated longer duration as compared to other studies as all patients stayed on ART for less than 3 years in Bedelle [15] and in Addis Ababa about 98% of patients stayed on ART for less than one and half years and in Dessie only 6% patients stayed on ART for more than 2 years [10, 16].

The main reason for initial ARV drug switch in the present study was toxicity which was known as peripheral neuropathy and it accounted for more than 60% of HAART regimen change. This finding was in agreement with the study conducted in some parts of Ethiopia [10]. The other reasons for HAART regimen change were hepatotoxicity 22(9.1%), anemia 16(6.6%) and rash15 (5.3%). The combined sum of hepatotoxicity, anemia and peripheral neuropathy was more than 80%, which was much higher than the studies done in United Kingdom (35%) [17] and India (27%) [18]. However, it was almost similar to the studies conducted in other parts of Ethiopia as it was 75.8% in Mekelle [19] and 66% in Dessie [16]. But there was a significant heterogeneity on the type of toxicities in these studies.

Change of the entire regimen from first-line to second-line is required in case of treatment failure. In order to increase likelihood of treatment success and minimize the risk of cross-resistance the new second-line regimen should involve drugs that keep activity against the patient’s virus strain and should preferably include at least three new drugs, one or more from a new class [12]. The preferred strategy for second-line ART for adults is using a boosted PI and two NRTI combinations when NNRTI-containing regimens were used in first-line ART [8]. Patients from low-income countries were less likely to change two or more drugs and to change to a protease-inhibitor-containing regimen when compared with patients from high-income countries [20].

The study also found that there was high prevalence of TB as almost three out of five patients (57%) had received isoniazid & cotrimoxazole prophylaxis and more than half (52%) of the patients had been treated for TB [16, 19]. NVP has high interaction with Rifampicin which is strong liver enzyme (CYP 3A4) inducer where by therapeutic level of NVP is decreased up to 40% which necessitates switch to EFV. Additive hepatotoxicity effects also exists when NVP and Rifampicin were used together according to findings of some studies which is another requirement to switch from NVP to EFV as the latter has lesser adverse drug reaction with Rifampicin [21].

## Conclusions

The number of patients who changed ARV drug in our resource constrained setting present a challenge to the restricted treatment choices that we currently own. The main reasons for ART switch were toxicity among which Peripheral neuropathy and hepatotocity were the leading toxicity for ART switch. Less toxic and better-tolerated HIV treatment options should be available and used more frequently in east and west Wollega zone health institutions. Patient should be evaluated regularly after a treatment change to assess for potential concerns with the new regimen, medication tolerance and to assess the effectiveness. Information is needed on patterns of resistance across the population to recommend future therapy options. Therefore national and health institution based surveillance of antiretroviral drug resistance should be conducted. It helps to know the resistance pattern and select the locally effective treatments. National level study on reasons for regimen change should be done to help drug suppliers and policy makers to improve and solve the problem. The reasons of ARV drug switch observed in this cross sectional study should be investigated further in longitudinal multicenter studies of ART utilization.

## Abbreviations

3TC: Lamivudine
ABC: Abacavir
ART: Antiretro Viral Therapy
ARV: Antiretroviral
ATV/R: Atazanavir/Ritonavir
AZT: Zidovudine
CD4: Cluster of Difference
CNS: Central Nervous System
CYP: Cytochrome p-450
d4T: Stavudine
ddI: Didanosine
EFV: Efavirenz
FTC: Emitricibine
HAART: Highly Active Antiretroviral Therapy
LPV/R: Lopinavir/Ritonavir
NNRTI: Nucleoside Reverse Transcriptase inhibitor
NRTI: Nucleoside Reverse Transcriptase inhibitor
NVP: Nevirapine
OI: Opportunistic Infection
PI: Protease Inhibitors
PLWHA: People Living With HIV/AIDS
SD: Standard deviation
TB: Tuberculosis
TDF: Tenofovir
VL: Viral Load
WHO: World Health Organization
